# Advances in Optical Coherence Tomography Imaging Technology and Techniques for Choroidal and Retinal Disorders

**DOI:** 10.3390/jcm11175139

**Published:** 2022-08-31

**Authors:** Joshua Ong, Arman Zarnegar, Giulia Corradetti, Sumit Randhir Singh, Jay Chhablani

**Affiliations:** 1 Department of Ophthalmology, University of Pittsburgh School of Medicine, Pittsburgh, PA 15213, USA; 2 Department of Ophthalmology, Doheny Eye Institute, Los Angeles, CA 90095, USA; 3 Stein Eye Institute, David Geffen School of Medicine at the University of California, Los Angeles, CA 90033, USA; 4 Nilima Sinha Medical College & Hospital, Rampur 852113, India

**Keywords:** optical coherence tomography, retina, choroid, advances, technology

## Abstract

Optical coherence tomography (OCT) imaging has played a pivotal role in the field of retina. This light-based, non-invasive imaging modality provides high-quality, cross-sectional analysis of the retina and has revolutionized the diagnosis and management of retinal and choroidal diseases. Since its introduction in the early 1990s, OCT technology has continued to advance to provide quicker acquisition times and higher resolution. In this manuscript, we discuss some of the most recent advances in OCT technology and techniques for choroidal and retinal diseases. The emerging innovations discussed include wide-field OCT, adaptive optics OCT, polarization sensitive OCT, full-field OCT, hand-held OCT, intraoperative OCT, at-home OCT, and more. The applications of these rising OCT systems and techniques will allow for a closer monitoring of chorioretinal diseases and treatment response, more robust analysis in basic science research, and further insights into surgical management. In addition, these innovations to optimize visualization of the choroid and retina offer a promising future for advancing our understanding of the pathophysiology of chorioretinal diseases.

## 1. Introduction

Optical coherence tomography (OCT) is an imaging modality that has revolutionized the field of ophthalmology. As a non-invasive imaging technique, OCT utilizes light and light interference to capture high resolution, cross-sectional tomographic information of biological tissue such as the retina and choroid at the micron level. This technology was first introduced in 1991 [1] and has been rapidly adopted into clinical practice in retina. Diagnostic evaluation in retinal and choroidal diseases are often conducted with OCT, including neovascular age-related macular degeneration (AMD) [2], central serous chorioretinopathy (CSCR) [3], vascular retinal disorders [4], and other vitreoretinal disorders [5]. OCT biomarkers have also been instrumental in further understanding and monitoring chorioretinal disease status; these biomarkers include central macular thickness, subretinal/intraretinal fluid, neurosensory detachment height, subfoveal choroidal thickness, choroidal vessel diameter, and choroidal vascularity index [6,7,8]. More recently, in the attempt to optimize the design of early interventional clinical trials for non-neovascular AMD, a number of structural OCT biomarkers, such as intraretinal hyperreflective foci, subretinal drusenoid deposits, drusen with hyporeflective core, high central drusen volume, have been described as high-risk for AMD progression to late stages [9,10,11,12].

Time-domain OCT (TD-OCT) was the first OCT system introduced to the world of clinical ophthalmology [13,14]. Compared to current systems, TD-OCT had a relatively slow scanning speed of 400 axial scans (A-scans)/second. Rapid advances allowed for increased axial resolution and scanning speed to optimize evaluation of the retina and choroid. For example, spectral-domain (SD) OCT and swept-source (SS) OCT were developed after TD-OCT and have acquisition times ranging from 27,000 A-scans/second to 100,000 A-scans/second. Axial resolution also increased, from around 10 μm with TD-OCT to 2 μm with SD-OCT and SS-OCT [13]. 

The advances in OCT technology have strengthened the ability to detect and diagnose for retinal disorders, often leading to earlier interventions and preservation of vision. OCT and OCT angiography (OCTA) of the retina have also been found to visualize and quantify the structure and the microvasculature of the retina. Moreover, enhanced penetration provides details of choroidal vasculature not previously seen with TD-OCT. Researchers have visualized sites of penetration of short posterior ciliary arteries and in eyes with thin choroid (i.e., especially highly myopic eyes) even the scleral vessels, posterior episcleral tissue, and Tenon’s layer can be delineated [15]. In addition, their applications are not limited to the retina field. In fact, OCT and OCTA imaging have been revolutionary in the field of glaucoma and neuro-ophthalmology by helping with early diagnosis of neurodegenerative diseases, including Alzheimer’s disease and possibly preclinical Alzheimer’s [16]. Thus, advances in OCT technology are promising in various fields of ophthalmology and neurology for earlier detection of the diseases, which may potentially improve the design of early interventional clinical trials. Given the wide application and evolving OCTA technology, including all OCTA advances is out of the scope of this article. In this paper, we review advances in OCT technology and techniques including wide-field OCT, visible light OCT, adaptive optics OCT, polarization-sensitive OCT, high-resolution OCT, intraoperative OCT, and handheld OCT. The emerging innovations made in this imaging modality will help advance several critical aspects in retinal care including imaging acquisition times, field of view, portability/accessibility, and intraoperative management.

## 2. Recent Advances in OCT Technology and Techniques

In this section and the following sections, we discuss the recent advances in OCT technology and techniques. Many of these techniques help to address current limitations to this clinically useful imaging modality. We organize these advances into three categories: (1) emerging advancements for clinical use, (2) advancements at the basic science/research level, and (3) recent advancements in currently available technology (Table 1). As these advances continue to progress in the future, these technologies will likely become more integrated and applied in the clinical setting. 

## 3. Visible Light OCT (Vis-OCT)

**Figure 1:**
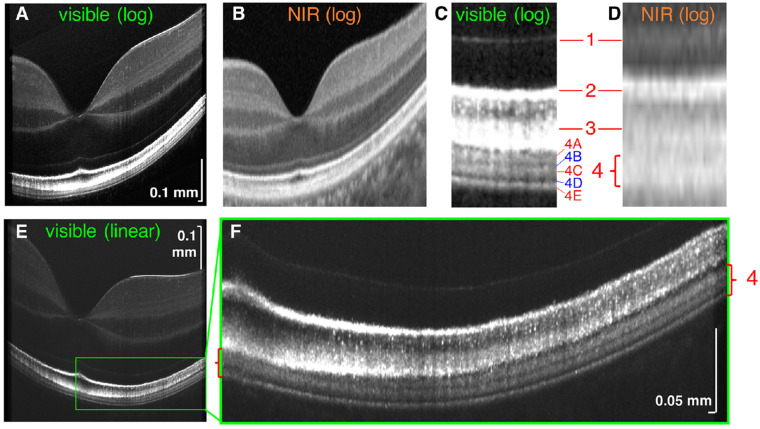
Visible light OCT (vis-OCT) imaging. Side-by-side comparison of vis-OCT (**A**) and commercial NIR-based OCT (**B**). (**C**) Magnified vis-OCT that shows outer retinal bands 1–4, with segmented hyperreflective bands and hyporeflective zones in outer retinal band 4, compared to magnified commercial NIR-based OCT (**D**). (**E**,**F**) Vis-OCT (linear scale). Reprinted with permission from Zhang et al. [18]. Visible Light Optical Coherence Tomography (OCT) Quantifies Subcellular Contributions to Outer Retinal Band 4. *Transl. Vis Sci. Technol.* 2021; 10(3): 30. with license permissions obtained from Creative Commons; Creative Commons Attribution 4.0 International License (CC BY 4.0, https://creativecommons.org/licenses/by/4.0/legalcode accessed on 1 August 2022).

Visible light OCT (vis-OCT) utilizes visible light for OCT illumination, rather than near-infrared (NIR) light, to capture images [33]. This technique allows for improved resolution of biological features of the retina due to shorter illumination wavelengths [33]. Zhang et al. recently reported the utilization of vis-OCT to quantify subcellular reflectivity contributions to the outermost retinal hyperreflective bands (Figure 1) [18]. Vis-OCT was first described by Povazay et al. [17]. Using a sub-15fs Ti:sapphire laser and photonic crystal fibers, this group demonstrated light emission in the range of 535 nm to 700 nm of the electromagnetic spectrum that improved axial resolution to <2 microns. This was achieved with a smaller bandwidth compared to current OCT illumination methods such as NIR. While most OCT devices currently utilize light in the NIR range because of its tissue penetration and reduced cost, there has been increasing interest in using vis-OCT [17]. Primary uses of vis-OCT are currently in blood vessel oximetry and imaging of healthy eyes [34,35].

Vis-OCT systems predominantly rely on supercontinuum lasers, which intrinsically generate relative intensity noise that restricts their clinical utility. Relative intensity noise can be attenuated by lengthening the camera’s exposure time, which subjects patients to excess light thus increasing their eye movements and hindering quality image acquisition. Rubinoff et al. proposed a balanced-detection vis-OCT model that uses two spectrometers to reduce relative intensity noise and tested it in a phantom retina and in vivo in human patients [36]. Results from their study indicated there may be a reduced need for exposing patients to excess light when employing balanced detection. The study results are unique as their method demonstrates more significant levels of relative intensity noise reduction than in previous setups. 

Speckle noise, which is often caused by the scattering of light waves, can negatively impact image interpretation in vis-OCT. Multi-volume image registration and modulation of B-scans have been suggested to reduce speckle noise [37,38]. The resultant improvement in vis-OCT image quality enhanced the visualization of neurons throughout all layers of rat retina. It allows vis-OCT to rival the capabilities of NIR AO-OCT. In particular, vis-OCT can now image structures such as the inner plexiform layer, the retinal pigment epithelium, and Bruch’s membrane [18,39]. Limitations to vis-OCT include depth-dependent dispersion limiting image quality. Zhang et al. demonstrated that water wavenumber calibration eliminates additional resampling steps and corrects dispersion [40]. 

## 4. Adaptive Optics (AO) in OCT (AO-OCT)

**Figure 2:**
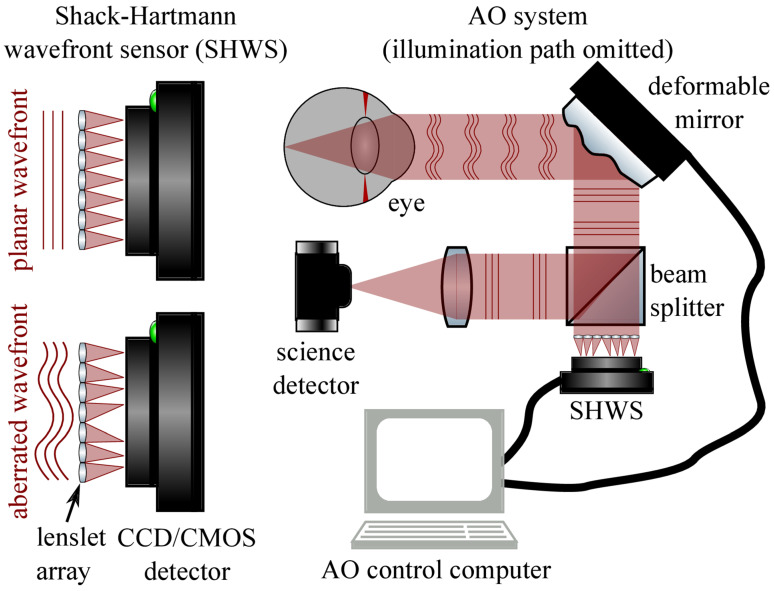
Adaptive optics technology system with Shack–Harmann wavefront sensor (SHWS) and deformable mirror schematic. SHWS utilizes a small lenslet array and samples a wavefront; displacements due to aberrations can drive a corrector (e.g., deformable mirror). This technology can help to visualize individual cells in the human retina [42,45]. Reprinted with permission from Jonnal et al. [45]. A Review of Adaptive Optics Optical Coherence Tomography: Technical Advances, Scientific Applications, and the Future. *Invest Ophthalmol. Vis Sci.* 2016; 57(9): OCT51-68 with license permissions obtained from Creative Commons; Creative Commons Attribution-Non-Commercial-No Derivatives 4.0 International (CC BY-NC-ND 4.0, https://creativecommons.org/licenses/by-nc-nd/4.0/legalcode (accessed on 1 August 2022)).

AO was initially developed to reduce dynamic wave-front errors in astronomical imaging [41]. It has since been found to quantify and eliminate high-order monochromatic aberrations from light passing through ocular tissues such as the cornea and lens. These aberrations cause poor lateral resolution in ophthalmic imaging and previously limited the clinical applications of various ophthalmic imaging systems [42,43]. AO systems are composed of a wavefront sensor (generally a Shack-Hartmann wavefront sensor) that measures distortions, and a wavefront corrector (typically a deformable mirror) that alters its shape to cancel out aberrations, and a controller that connects these elements (Figure 2) [44,45]. 

**Figure 3:**
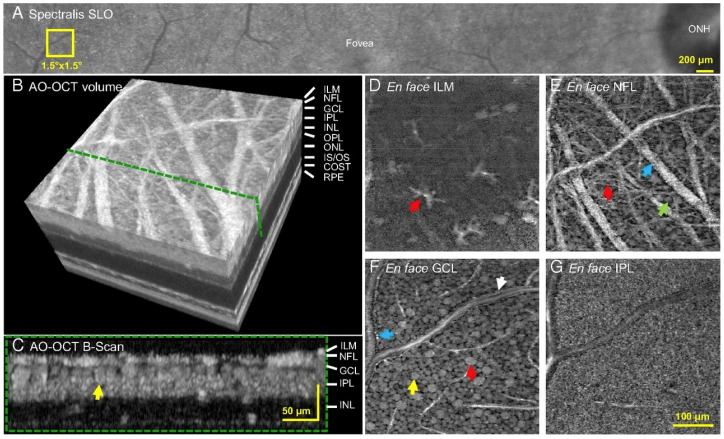
Adaptive optics (AO) OCT showcasing cellular structures of the retina. Yellow 1.5 × 1.5 box in (**A**) (Spectralis scanning laser ophthalmoscope) showcases location imaged by AO-OCT. (**B**) 3D AO-OCT with layers and green dotted line showcases the cross-section of the retina in (**C**) with yellow arrow highlighting ganglion cell layer soma. (**D**–**G**) Different layers of the retina (internal limiting membrane, nerve fiber layer, ganglion cell layer, and inner plexiform layer). (**D**) Red arrow shows astrocyte/microglial cells. (**E**) Blue arrow shows nerve fiber webs. (**F**) Red arrow shows large soma, yellow arrow shows ganglion cell layer soma, blue and white arrows show edges of vessel walls. (**G**) Synaptic connections in the internal plexiform layer. Reprinted with permission from Liu et al. [50]. Imaging and quantifying ganglion cells and other transparent neurons in the living human retina. *Proc. Natl. Acad. Sci. USA* 2017; 114(48): 12803-8 with license permissions obtained from Creative Commons; Creative Commons Attribution-Non-Commercial-No Derivatives 4.0 International (CC BY-NC-ND 4.0, https://creativecommons.org/licenses/by-nc-nd/4.0/legalcode (accessed on 1 August 2022)).

Arguably the most impactful feature of AO in ophthalmology is that it permits the imaging of individual cells, such as photoreceptors, in vivo [42,46,47]. AO has a lateral resolution of 2 microns, a considerable improvement from the ~15-micron lateral resolution of OCT [48]. Initially, AO was used with en-face imaging modalities to demonstrate individual rods and cones in 2D. When combined with OCT, AO allows for 3D imaging and the resolution of structures such as photoreceptors and the retinal pigment epithelium. Efforts have been made to increase the FOV of AO-OCT systems in imaging these cells, increasing the area from ~1 degree to 4 degrees × 4 degrees (Figure 3) [49].

Computational AO (CAO) reduces aberrations by modifying the phase of OCT data in the spectral domain and has been studied with much fervor in recent years. While image quality tends to be sacrificed with CAO, it was designed to reduce the need for costly hardware by correcting distortions after data collection [44,51]. The primary CAO modality today is interferometric synthetic aperture microscopy (ISAM), a computational imaging technique that enhances depth-independent resolution [52]. ISAM requires limited movement from the patient for optimal imaging quality. A stretched-pulse mode-locked laser light source was tested to increase the A-scan rate and combat the adverse effects of eye movement [53]. Boppart et al. established the first model of CAO in polarization-sensitive OCT (PS-OCT), which corrects low-order aberrations in ex vivo human tissues [54]. There have also been proposed advances in improving CAO aberration correction capabilities and image quality [55]. CAO may streamline image collection workflows and promote cost-savings in the clinic, though with loss of image quality.

Sensorless AO (SAO) is another alternative to hardware-based AO-OCT that relies on the images’ properties rather than a wavefront sensor to ultimately measure and correct aberrations [56]. SAO optimization methods and algorithms include Zernike Mode Hill Climbing [57], stochastic parallel gradient descent [58,59], deep reinforcement learning [60], and others. SAO features have been tested to some extent in CAO models as well [58,61]. Since its description 15 years ago, AO-OCT is still not commonly used in ophthalmology clinics due to certain limitations. High magnification images are prone to motion artifacts and require constant fixation [62]. This becomes increasingly difficult in eyes with AMD-related geographic atrophy, retinal dystrophy such as cone dystrophy. Moreover, poor mydriasis, or presence of any media opacity significantly affect the quality of the images [62]. Other concerns are related to the very high acquisition cost, lack of commercial interest, need for trained manpower, and availability of ample space to house AO-OCT. Despite these limitations, researchers and clinicians can identify and broaden the clinical utility of AO-OCT in AMD, diabetic retinopathy, inherited retinal dystrophies, and other specialties such as glaucoma. 

## 5. Polarization-Sensitive (PS) OCT

First demonstrated in 1992, PS-OCT functions by analyzing the polarization state of backscattered light and measuring birefringence in the tissue sample. Different tissues can change the polarization state of the OCT light source [63]. Initial PS-OCT schemes were based on TD-OCT. However, PS is now employed in both SS-OCT and SD-OCT to image various ocular structures such as the macula and peripheral retina [21,64,65]. A challenge in recognizing AMD early is detecting drusen; PS-OCT can be used to segment the RPE and identify drusen [66]. In many fiber-based PS-OCT setups, the laser light is initially polarized, then the optical fiber is fixed to prevent changes in the polarization state. Any changes to the optical fiber after that would affect the polarization state of the light source. A variation of SS-based PS-OCT was tested that uses a depolarizer and a polarizer to achieve a model independent of the input polarization, albeit with a drop in sensitivity and considerable loss of input light [67]. Another technique minimizes changes in the polarization state of the incident light beam by using a common-path interferometer in conjunction with polarization-maintaining fibers to promote stability of the optical fiber [68]. 

Adaptations of PS-OCT include polarization-sensitive quantitative OCT (PS-QOCT). QOCT provides dispersion-cancellation and identifies the refractive index of a media, and when combined with PS, offers improved resolution compared to traditional methods [69,70]. In a recent study, Sukharenko et al. demonstrated imaging and characterization of a birefringent material using PS-QOCT, which may have future applications in imaging biological tissues [71]. 

**Figure 4:**
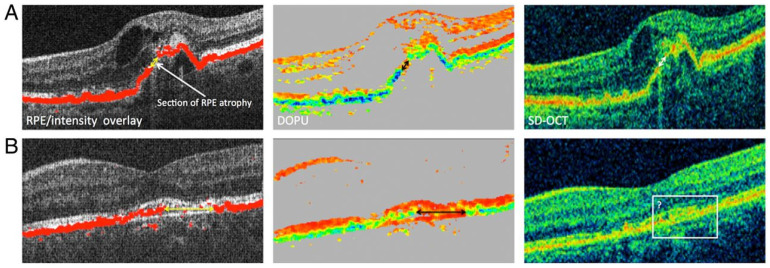
Polarization-sensitive optical coherence tomography (PS-OCT). (**A**) Three-figure panel showcases a comparison of PS-OCT (**top middle**) and SD-OCT (**top right**) where both modalities are able to identify retinal pigment epithelium atrophy. (**B**) Three-figure panel showcases a comparison of PS-OCT (**bottom middle**) and SD-OCT (bottom right) where PS-OCT can more clearly identify the retinal pigment epithelium atrophy. Reprinted with permission from Schütze, C et al. Polarisation-sensitive OCT is useful for evaluating retinal pigment epithelial lesions in patients with neovascular AMD. *British Journal of Ophthalmology* 2016; 100: 371–377 with license permissions obtained from Creative Commons; Attribution-NonCommercial 4.0 International (CC BY-NC 4.0, https://creativecommons.org/licenses/by-nc/4.0/legalcode (accessed on 1 August 2022)).

PS-OCT carries many promising applications in both basic and clinical ophthalmic research, particularly in automated segmentation of retinal structures such as RPE [66]. Similarly, geographic atrophy commonly seen in the dry form of AMD can be segmented using PS-OCT [21]. Fibrotic tissues, which contain collagen, are particularly birefringent and are thus imaged well by PS-OCT. Schütze et al. demonstrated that PS-OCT was useful for the evaluation of RPE lesions in choroidal neovascularization in eyes with neovascular AMD (Figure 4) [72]. Retinal fibrosis growth in the setting of neovascular AMD can be tracked using PS-OCT and segmentation algorithms [73].

## 6. High-Resolution OCT (High-Res OCT)

One of Heidelberg Engineering’s recent developments is the introduction of high-resolution OCT (High-Res OCT). High-Res OCT increases the bandwidth of the OCT light source which allows for an increase in optical axial resolution [23]. High-Res OCT is capable of 3 µm axial resolution, allowing for capturing clearer images of the small vasculature, including the choriocapillaris [23,24]. The choriocapillaris has been found to play a role in many retinal diseases, thus more detailed visualization of this microvasculature will likely advance understanding of its dysfunction in these diseases [74]. Spaide and Lally reported the utilization of High-Res OCT to evaluate a patient with multiple evanescent white dot syndrome (MEWDS) [24]. Their investigation with this OCT imaging of up to 3 µm axial resolution suggested that the interdigitation zone (IZ) showed persistent abnormalities in this patient with MEWDS rather than the ellipsoid zone (EZ), the primary zone of involvement noted in previous MEWDS studies [24]. The utilization of this advancement in OCT imaging may help to provide additional insight into the microstructures and microvasculature of the retina in chorioretinal diseases.

## 7. Full-Field OCT (FFOCT) and Dynamic FFOCT (D-FFOCT)

**Figure 5:**
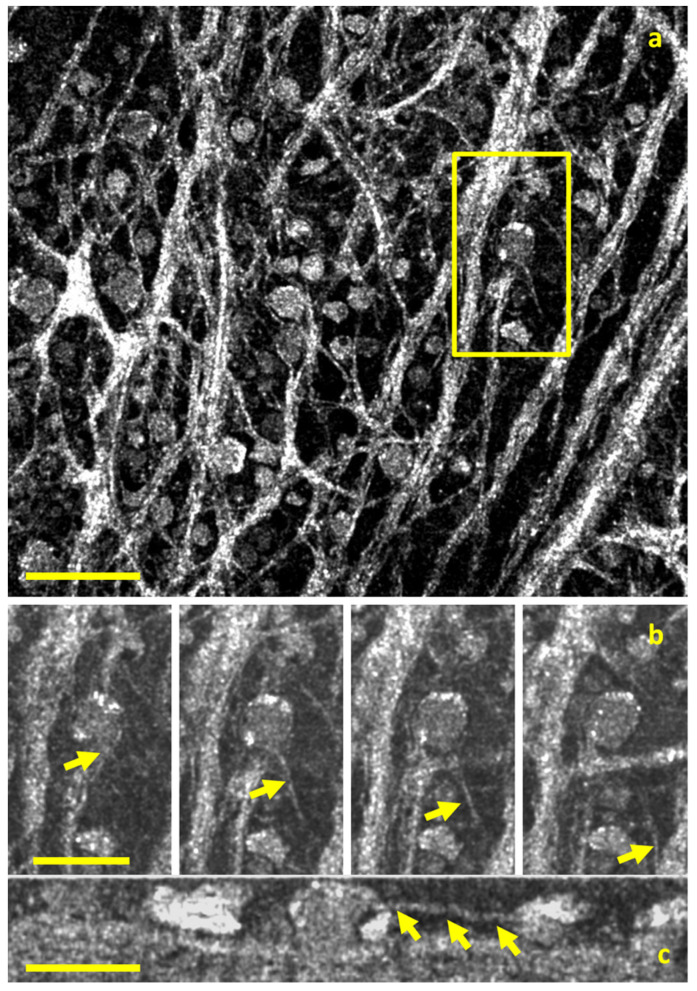
Full-field optical coherence tomography (FF-OCT) imaging the human retina. (**a**) The en face view of the human nerve fiber layer (scale bar is 500 μm). (**b**) A four-panel image of a 2 μm thick axon (yellow arrow) moving away from a ganglion cell soma. (**c**) A cross-section the same cell in (**b**) along the length of the axon (scale bar is 50 μm). Reprinted with permission from Grieve et al. [25]. Appearance of the Retina With Full-Field Optical Coherence Tomography. Invest. Ophthalmol. Vis. Sci. 2016; 57(9): OCT96–OCT104 with license permissions obtained from Creative Commons; Creative Commons Attribution-NonCommercial-NoDerivatives 4.0 International (CC BY-NC-ND 4.0, https://creativecommons.org/licenses/by-nc-nd/4.0/legalcode (accessed on 1 August 2022)).

Full-field OCT (FFOCT) captures 2D enface scans of ocular tissue at different depths. These can be used to reconstruct 3D volumetric images with resolutions of up to 1 micron. The setup most commonly relies on incoherent illumination and a Linnik interferometer, with two microscope objectives in the reference and sample arms. FFOCT possesses clinical value as an optical microscopy tool because it can capture subcellular structures for tissue examination in a non-invasive, efficient manner. Its current utilization has bolstered basic science research in cellular-resolution analysis. Current research has employed FFOCT to visualize detailed aspects of the human retinal ganglion cell axons [25] (Figure 5).

A derivative of FFOCT has been described that similarly provides visualization of dynamic structures at the microscopic level. In D-FFOCT, backscattered light from subcellular structures in motion can be measured in a time-dependent fashion that potentiates live or time-lapse imaging [75]. Like FFOCT, D-FFOCT primarily utilizes incoherent light and a Linnik interferometer, though without a reference arm [76]. When used in conjunction with fluorescence microscopy, histological techniques, or multimodal setups, highly specific structures can be marked, identified, and examined in situ. A full-field form of confocal microscopy, structured illumination microscopy, has been used with dynamic and static FFOCT methods [77]. 

A recently described application of D-FFOCT is in the 3D imaging of retinal organoids (ROs). Derived from human-induced pluripotent stem cells, ROs are tissues that form 3D structures such as the developmental optical vesicle and optic cups and, ultimately, the retina. The design and implementation of ROs have been ground-breaking in ophthalmologic research because it closely mimics the structure and functionality of the human retina. Areas of study that benefit from using ROs include retinal transplantation [78,79], drug delivery, optic nerve diseases [80], and others. 

Scholler et al. introduced a novel method of label-free imaging of retinal organoids using D-FFOCT and used it to monitor the temporal development of ROs with a temporal resolution of 20 ms [81]. The authors showed that the metabolic activity of cells could be determined and used to differentiate cells, such as those undergoing apoptosis and rapidly dividing. Validated by multimodal imaging that overlayed fluorescence with D-FFOCT images, the success of this method suggests an imaging system that can identify specific without the need for exogenous dyes or antibodies is on the horizon. Groux et al. recently presented findings from an experiment in which porcine RPE cells and human-induced pluripotent stem cell-derived RPE were captured with live D-FFOCT imaging before and after exposure to toxic stress [82]. The authors explored the dynamics of intracellular organelles during wound healing and showcased a semi-automatic segmentation-based software (SAVE Profiler) that segmented the RPE wound and determined its dimensions. Their results indicate that D-FFOCT and the SAVE Profiler may have applications in diseases of the RPE, such as age-related macular degeneration.

In summary, FF-OCT offers 2D en face imaging of ex vivo tissues with resolution that is quickly approaching that seen in histological sample preparation. Its in vivo use is made difficult due to motion artifact. The study of ROs has potential to model human retinal in health and disease, and D-FFOCT now offers a means of imaging individual cells in situ to follow retinal development. 

## 8. Wide-Field and Ultrawide-Field OCT (WF-OCT and UWF-OCT)

**Figure 6:**
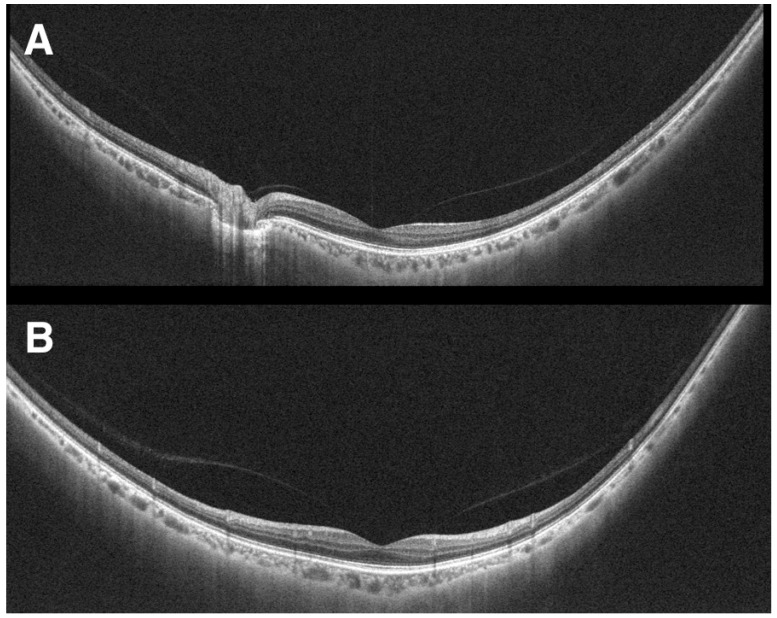
Ultrawide-field optical coherence tomography (UWF-OCT) Image. (**A**) Horizonal scan image (23 mm in length). (**B**) showcases vertical scan (20 mm in length). Reprinted with permission from Takahashi et al. [28]. Ultra-Widefield Optical Coherence Tomographic Imaging of Posterior Vitreous in Eyes With High Myopia. *Am J Ophthalmol.* 2019; 206: 102-12. with license permissions obtained from Elsevier and Copyright Clearance Center.

**Figure 7:**
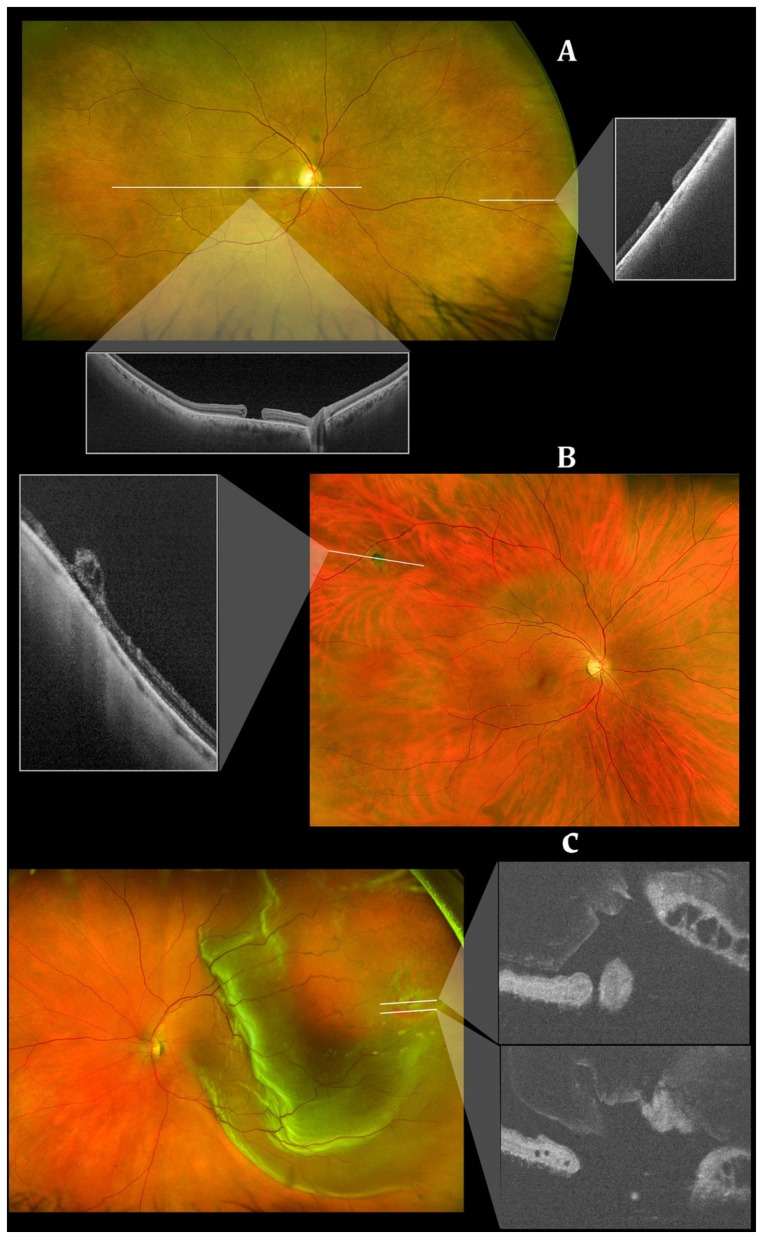
Integrated scanning laser ophthalmoscope and ultra-widefield imaging for peripheral optical coherence tomography with Optos’ Silverstone swept-source optical coherence tomography (Optos PLC, Dunfermline, UK). (**A**) A peripheral atrophic retinal hole (right rectangle) and macular hole (lower rectangle). (**B**) A cystic retinal tuft in the peripheral retina. (**C**) A retinal detachment in the peripheral retina. Reprinted with permission from Sodhi et al. [96]. Feasibility of peripheral OCT imaging using a novel integrated SLO ultra-widefield imaging swept-source OCT device. Int Ophthalmol 2021; 41(8): 2805-15 with license permissions obtained from Creative Commons; Creative Commons Attribution 4.0 International License (CC BY 4.0, https://creativecommons.org/licenses/by/4.0/legalcode (accessed on 1 August 2022)).

Although a powerful ocular imaging tool, OCT imaging is often limited with a relatively narrow field of view (FOV). The FOV is usually constrained to around 20 degrees × 20 degrees [83]. Fundus cameras such as the Pomerantzeff equator-plus camera [84] and ultrawide-field scanning laser ophthalmoscopy (SLO) [85] addressed the issue of narrow FOV but only produced 2D scans. To address this gap in imaging capability, wide-field OCT technology (WF-OCT) with FOV around 40–55 degrees and ultrawide-field OCT (UWF-OCT) with FOV up to 200 degrees in a volumetric scan were developed (Figure 6) [28,86]. SS-OCT with a higher imaging speed (100,000 A scans/s) also has an advantage of enhanced depth scan range which is essential to image the curved contour of the peripheral retina [87]. Early WF-OCTs were based upon an InGaAs diode array that enabled a higher readout rate and a FOV of 38 degrees [27]. The initial UWF-OCT system described by Klein et al. [88] is based upon ultrahigh speed swept source (SS) OCT that employs a 1050 nm Fourier domain mode locked laser. It can produce 1900 × 1900 A-scans with a 70–degree FOV within three to six seconds. In 2018, Gresores et al. demonstrated that a prototype multimodal system that combined ultrawide-field SLO and OCT could provide similar visualization of retinal structures to standalone OCT yet allowed for observation of additional lesions outside the OCT scanning field [89]. At present, commercially available WF OCT machines include the NIDEK Mirante^®^ (NIDEK Co. Ltd., Gamagori, Japan) high definition SLO/OCT model which includes an adapter that allows for 163 degree UWF imaging [90,91]. Another system is Heidelberg Engineering’s Spectralis^®^ OCT (Heidelberg Engineering, Heidelberg, Germany) which utilizes a similar multimodal SD-OCT with confocal scanning laser ophthalmoscope to generate a FOV of up to 55 degrees. This system can be combined with the Ocular Staurenghi 230 SLO Retina Lens to produce a FOV of 150 degrees [92]. The Optos’ Silverstone^®^ (Optos PLC, Dunfermline, UK) integrates scanning laser ophthalmoscope and UWF imaging with SS-OCT for a 200-degree single-capture image in less than 0.5 s [93,94]. Another Optos OCT device, the Optos’ Monaco^®^ (Optos PLC, Dunfermline, UK), integrates UWF imaging with SD-OCT for a 200-degree single-capture image in less than 0.5 s [95]. Integration of these advances to capture the peripheral retina allows for peripheral OCT of retinal diseases including retinal tears, retinal holes, retinoschisis, retinal tuft, lattice degeneration, CSCR, choroidal nevi, and choroidal lesions (Figure 7) [96].

Experimental methods of widening the FOV of OCT include extended field imaging (EFI). Described by Uji et al. [97], EFI utilizes swept-source OCT with a +20.00-diopter lens between the eye and the OCT probe to increase the FOV to nearly 60 or 70 degrees. This offers a simple way to achieve wider FOV for imaging the periphery. A recent study swapped the +20.00-diopter lens for a +90.00-diopter double aspheric noncontact slit-lamp lens in a swept-source OCT system. This method was dubbed “innovative wide-field” OCT. In the study, innovative wide-field OCT was compared to standard 12 mm OCT in imaging the retina in 50 eyes of 25 patients with proliferative diabetic retinopathy. Innovative wide-field technology increased the scan length by a factor of 1.65 ± 0.67; however, this setup had more rim and edge artifacts and poorer image quality compared to standard OCT [98].

Mori et al. described a technique that combines multiple SD-OCT scans of the posterior vitreous cortex and vitreoretinal interface into a montage of images that mimics WF-OCT [99]. The montage can be achieved by obtaining multiple scans with the subject focusing on different targets, then combining the images via image editing software. This methodology has been adopted to increase scan sizes in OCT angiography (OCTA) as well. Though it improves the visualization of microvasculature in the peripheral retina, montaging is susceptible to distortions, low-OCT-signaling, reduced sampling density, and other artifacts negatively impacting its clinical utility [100]. 

WF-OCT provides additional information compared to the routine 6–9 mm scans in conditions such as DR, CSCR, polypoidal choroidal vasculopathy (PCV), peripapillary choroidal neovascular membrane (CNVM) or uveitic entities. Anatomical details of peripheral retinal changes such as ischemic areas in DR, retinal vein occlusions, or site of retinal breaks, peripheral retinal detachment, retinoschisis and choroidal lesions (melanoma, nevus, hemangioma, choroidal metastasis) can be easily obtained. 

Artificial intelligence (AI) has played an increasingly important role in optimizing delivery of care and research in ophthalmology [101]. As such, this powerful technology has been applied to WF SS-OCT imaging for retinal diseases. Deep learning (DL), especially convolutional neural networks (CNN), have been implicated in studying age-related macular degeneration (AMD) progression. A prominent challenge in utilizing AI in understanding retinal disease is a general paucity of 3-dimensional (3D), volumetric scan data. A novel deep learning technique, SLIVER-net, can be trained on a dataset of 2-dimensional (2D) scans to predict AMD biomarkers (intraretinal hyperreflective foci, subretinal drusenoid deposits, and hyporeflective drusen cores) and risk factors in 3D volumes via transfer learning [102]. Zhang et. al. used deep-learning to automatically detect and quantify geographic atrophy in patients with AMD in 2D B-scans with success [103]. In this study, models were generated to identify features such as RPE loss that are used to grade geographic atrophy. As this technology continues to become further validated, the pairing of AI with OCT will likely help clinicians to monitor retinal diseases even more closely.

Wide field imaging techniques including WF-OCT are prone to certain challenges. Optical aberrations increase with increase in FOV manifesting as increased noise to signal ratio [104]. Pupillary and ciliary shadowing related artifacts further reduce the image quality. Increased peripheral retinal curvature and inter-individual variation in retinal curvature and the need for very high A scan rate (>1 million A scan per second) are other variables which need to be addressed to obtain analyzable dense wide-field OCT scans. To summarize, WF-OCT and ultrawide-field OCT provide the clinicians with high quality, non-invasive, in-vivo tomographic details of chorioretinal layers and aid in the management of these chorioretinal disorders. 

## 9. Hand-Held and Intraoperative OCT (iOCT) 

Although a powerful imaging technology, commercially available OCTs are relatively large and typically table-mounted and with limited portability [105]. Hand-Held OCT technology has been developed to address these limitations and serve as an efficient and diagnostic point-of-care imaging tool [106]. One of the key utilizations noted for hand-held OCT is to help remove barriers to care for OCT imaging in the infant and young child population [107,108,109]. As commercial OCT systems are typically not designed for infants; the use of a portable OCT can help address this limitation in this OCT for identifying vision-threatening diseases in this patient population. In addition hand-held OCT can also address limitations for imaging bedridden patients [110]. Hand-Held OCT usually have two components: a lighter, hand-held piece, and a bulkier, base unit which contains the light source, reference arm, spectrometer, and computer with its display. An overall reduction in size of base unit, its constituents and transferring the interferometer to handpiece led to significant reduction in cost. Low-cost OCT therefore can be available at market prices of approximately 5000–7000 USD i.e., a reduction of >70% compared to the commercially available OCT devices [105,111]. Several challenges for a handheld OCT system include operator variability, hand movement, and manual alignment [112]. 

Widefield technology has also been integrated into handheld OCT imaging, as well as OCTA, increasing the field of view of this useful, portable technique [29]. Continued clinical validation is needed for this promising technology, and its development has served as a powerful catalyst for OCT utilization during vitreoretinal surgery [113].

**Figure 8:**
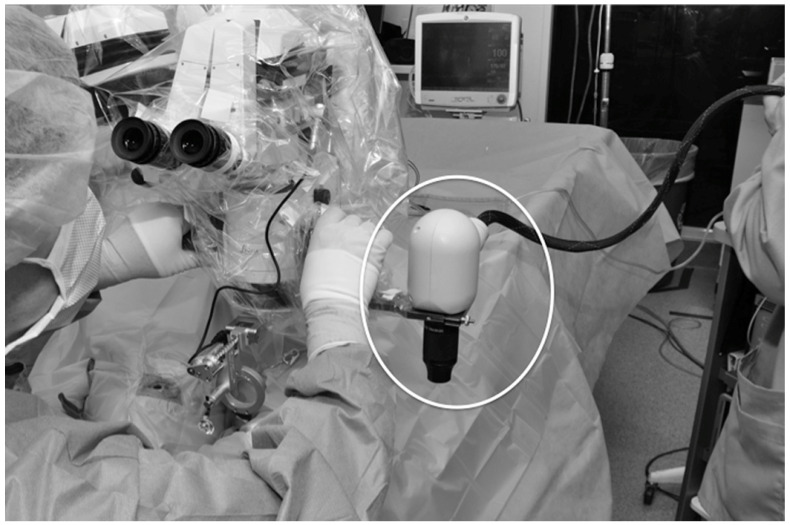
Intraoperative OCT with portable mounted microscope (circled) by Ehlers et al. [115]. Reprinted with permission from Ehlers et al. The Prospective Intraoperative and Perioperative Ophthalmic ImagiNg with Optical CoherEncE TomogRaphy (PIONEER) Study: 2-year results. *Am J Ophthalmol.* 2014 Nov; 158(5): 999–1007 with license permissions obtained from Elsevier and Copyright Clearance Center.

As a powerful imaging tool in the clinic, OCT imaging has been explored to provide additional visualization for ophthalmic surgeries [114]. Intraoperative OCT (iOCT) allows for surgeons to utilize microscope-integrated, OCT imaging in real-time for feedback and image guidance in the operating room (Figure 8) [115].

**Figure 9:**
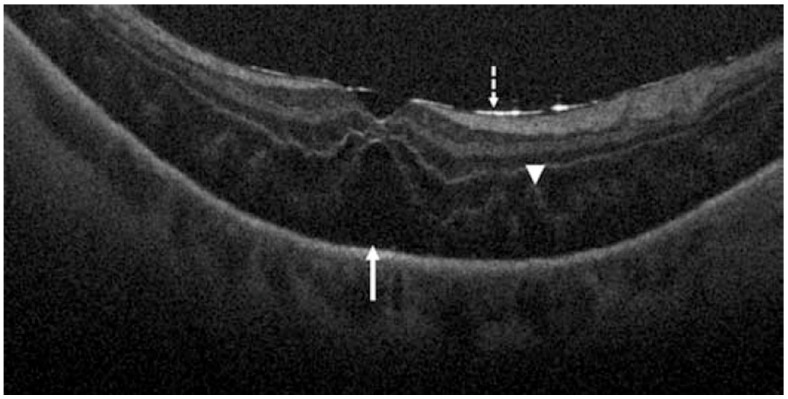
Retinal detachment visualized by intraoperative OCT. Dashed arrow shows hyperreflective retina and perfluorocarbon liquid interface, arrowhead shows outer retinal corrugations, and solid arrow shows persistent subretinal fluid. Reprinted with permission from Ehlers et al. [115]. The Prospective Intraoperative and Perioperative Ophthalmic ImagiNg with Optical CoherEncE TomogRaphy (PIONEER) Study: 2-year results. *Am J Ophthalmol.* 2014 Nov; 158(5): 999–1007 with license permissions obtained from Elsevier and Copyright Clearance Center.

The Prospective Intraoperative and Perioperative Ophthalmic ImagiNg with Optical CoherEncE TomogRaphy (PIONEER) and Determination of Feasibility of Intraoperative Spectral Domain Microscope Combined/Integrated OCT Visualization During En Face Retinal and Ophthalmic Surgery (DISCOVER) studies were started to investigate the utility of iOCT in various ophthalmic surgeries [115,116]. These studies included both anterior and posterior segment surgeries for preoperative diagnoses including epiretinal membrane, retinal detachment (Figure 9), vitreous hemorrhage, and vitreomacular traction [115]. Surgeries with iOCT included fluocinolone intravitreal implant and pars plana vitrectomy [115,116]. The DISCOVER study also included pars plana vitrectomy with combined iOCT and Ngenuity’s digital heads-up, 3-dimensional visualization system (Ngenuity, Alcon, Fort Worth, TX, USA) [117]. This digital integration with iOCT allowed for the surgeon to view an overlay of the OCT data on top of the surgical field on a 4K high-definition monitor. The digital system allowed for the surgeons to review OCT data without turning away from the surgical field, and the surgeons reported excellent image visualization and contrast [117].

Various systems have been developed and iOCT continues to be an area of high interest for optimizing surgical retinal care. Multiple iOCT options including hand-held portable probe, microscope mounted, and microscope integrated are available at present [118]. Microscope-integrated design helps to visualize the real-time vitreoretinal interface interaction with surgical instruments and corresponding changes intraoperatively. For instance, surgeons can assess intraoperative macular hole closure or identify remnants of epiretinal membrane during membrane peel. iOCT may also obviate the need to use dye staining during macular surgeries. iOCT may also facilitate the therapeutic delivery of drugs e.g., tissue plasminogen activator [119] or placement of electrodes array for subretinal implants [120]. Further research must be conducted to evaluate the widespread impact that iOCT has on vitreoretinal surgery [30]. 

## 10. At-Home OCT

Given the nature of certain retinal diseases (e.g., neovascular AMD), frequent monitoring via clinic visits and OCT imaging is required to ensure proper management [31]. These visits can often be burdensome, especially for the elderly population [121]. Notal Vision Home OCT, an at-home SD-OCT, allows for daily self-imaging for patients at risk for worsening retinal disease. Studies that have evaluated this at-home, self-imaging OCT technology have reported good agreement on retina biomarkers with scans from in-clinic OCT [31,32,121]. Liu et al. reported a prospective, longitudinal study with 15 participants with this technology and observed a mean daily self-imaging rate of 80% (or 5.7 scans per week) [121]. The consistent, near-daily monitoring of retinal and choroidal diseases may allow for precise treatment planning. Artificial intelligence has also been developed with the Notal OCT Analyzer to automate the identification of intra- and subretinal fluid [31,122]. While this technology will continue to undergo testing and further research prior to widespread adoption, the ability to have OCT scans done near-daily at home may help to prevent vision loss for many in the future.

## 11. Conclusions

OCT technology continues to progress to address certain limitations observed in the current standards of care for choroidal and retinal diseases. In addition, these advances will help to advance basic science research and our understanding of the pathophysiology of chorioretinal diseases. As evidenced by technologies such as ultrawide-field OCT, these applications can help to detect and monitor retinal diseases with OCT capabilities at the periphery. As observed with full-field OCT, this innovation allows for analysis up to the individual human retinal ganglion cell axon. As seen with OCT advances in currently available technologies, such as intraoperative OCT, these innovations allow for further insight into the surgical management of chorioretinal disorders. At-home OCT demonstrates the ability to bring this powerful technology to the homes of at-risk individuals for a new frontier of retinal monitoring. As future research continues to develop, the goal is for emerging, clinically validated OCT technology to become more widely adopted. These technologies represent a promising future in optimizing the understanding, diagnosis, monitoring, and management of diseases in retina.

## Figures and Tables

**Table 1:** Advances in optical coherence tomography (OCT) imaging technology and techniques. Advances are categorized into emerging advances for clinical use, advances for basic science/research, and recent advances in available technology.

Advances in OCT	Summary of Primary Advancement	References
	**Emerging Advances for Clinical Use**	
Visible-Light (Vis) OCT	Utilizes visible light illumination for OCT as opposed to commonly used near-infrared (NIR) light to capture fine details of the retina	[17,18]
Adaptive Optics (AO) OCT	Wavefront correcting component and computational controller software to compensate for aberrations and quality degradation, increasing the quality of OCT images.	[19,20]
Polarization Sensitive (PS) OCT	Measures and quantifies the polarization and depolarization of tissue for precision, high-quality imaging of retinal pigment epithelium layers	[21,22]
High-Resolution OCT (High-Res OCT)	Broadened bandwidth of the OCT light source to improve axial resolution and capture clearer details of the retinal microstructures and microvasculature.	[23,24]
	**Advances for Basic Science/Research**	
Full-Field (FF) and Dynamic Full-Field (DFF) OCT	Acquires images with charge coupled device cameras in 2D enface orientation at different depths for high resolution images at the cellular level.	[25,26]
	**Recent Advances in Available Technology**	
Wide-field (WF) and Ultrawide-field (UWF) OCT	Increased field of view to 40–55 degrees with wide-field OCT and up to 200 degrees with ultrawide-field OCT	[27,28]
Hand-Held and Intraoperative OCT (iOCT)	Hand-held OCT is portable OCT technology that is particularly useful for infants and bed ridden patients. Intraoperative OCT (microscope integrated) allows for image guidance and real-time feedback during ophthalmic surgery.	[29,30]
At-Home OCT	At-home, self-imaging OCT that allows for more frequent imaging and good agreement when compared to in-clinic OCT for more precise management of retinal diseases.	[31,32]
